# Sports foods are not all they shake up to be. An audit of formulated supplementary sports food products and packaging in Australian retail environments

**DOI:** 10.3389/fnut.2023.1042049

**Published:** 2023-02-14

**Authors:** Celeste I. Chapple, Catherine G. Russell, Alissa J. Burnett, Julie L. Woods

**Affiliations:** School of Exercise and Nutrition Sciences, Institute for Physical Activity and Nutrition, Deakin University, Geelong, VIC, Australia

**Keywords:** sports foods, food regulation, claims, marketing and advertising, nutrition

## Abstract

**Objective:**

To determine store availability, total number of products, and types of Formulated Supplementary Sports Foods in Australia, along with their stated nutrition content, sweeteners added, total number, and type of claims displayed on the packaging.

**Design:**

A cross-sectional, visual product audit of mainstream retailers.

**Setting:**

Supermarkets, pharmacies, health food stores, and gym/fitness centres.

**Results:**

A total of 558 products were captured in the audit, 275 of which displayed the correct mandatory packaging attributes. Three categories of products were identified, based on the dominant nutrient. Only 184 products appeared to display the correct energy value based on the listed macronutrient content (protein, fat, carbohydrate, dietary fibre). The stated nutrient content was highly variable across all product subcategories. Nineteen different sweeteners were identified, with most foods containing only one (38.2%) or two (34.9%) types. The predominant sweetener was stevia glycosides. Packages displayed multiple claims, with a maximum of 67 and minimum of 2 claims. Nutrition content claims were most frequently displayed (on 98.5% of products). Claims included regulated, minimally regulated and marketing statements.

**Conclusion:**

Sports food consumers should be provided with accurate and detailed on pack nutrition information, to ensure informed choices are made. However, this audit showed multiple products which did not conform to current standards, appeared to provide inaccurate nutrition information, contained multiple sweeteners, and displayed an overwhelming number of on-pack claims. The increase in sales, availability, and products available in mainstream retail environments, could be impacting both intended consumers (athletes), and general non-athlete population. The results indicate underperformance in manufacturing practices which preference marketing over quality and stronger regulatory approaches are needed to protect consumer health and safety, and to prevent misleading consumers.

## Introduction

For athletes, specialised nutrition is key to enabling optimum performance (1, 2). When nutritional needs for protein or carbohydrate are not met *via* diet, formulated food products can be used (3). The Australian Institute of Sport (AIS) states that sports foods can play a small but important role for high performance athletes and developed a classification system based on the level of scientific evidence to support their use in this group (4). Sports foods dominant in protein [powders/Ready-To-Drink (RTD) beverages/bars/snacks], carbohydrate (gels/powders) and some additional products such as beta alanine and creatine are permitted for use by identified athletes and considered as group A foods/supplements, with strong scientific evidence for use in specific situations (4). While most of the additional products available such as branched chain amino acids, glutamine, and pre workout are considered to have either not enough scientific evidence to support use or are banned due to contamination risk (4). Sports Integrity Australia recommends that no supplement is safe for athletes to consume and if they are to consume supplements that only batch tested products with the trusted by sport or Human And Supplement Testing Australia (HASTA) are consumed (4). These other sports foods generally contain one or two ingredients with specific physiological purposes such as stimulating muscle protein synthesis, or providing an ergogenic aid, such as caffeine, to enhance physical performance (2, 3).

In some countries these are regulated by different agencies for example, food supplements by the Food Standards Agency (United Kingdom) (5), foodstuffs, the European Commission (European Union) (6), dietary supplements, National Institutes of Health, Office of Dietary Supplements (United States) (7), and supplemented foods, the Government of Canada (Canada) (8). The regulations cover labelling for health or performance claims, marketing aspects in relation to the contents of vitamins, minerals, and any other substances, directions for use and safe consumption (5–9). In Australia, the location of the present study, these products are known as “*Formulated Supplementary Sports Foods*” (herein referred to as sports foods) and are regulated by Food Standards Australia New Zealand (FSANZ), under Standard 2.9.4 of the Food Standards Code (FSC) (10). Generally, products are not evaluated by the relevant agencies, prior to being made commercially available and it is the manufacturers’ responsibility to ensure that the benefits, safety, and compositional specifications are met (5, 7, 8, 11). Standard 2.9.4 also sets the permitted ranges of macronutrients, added vitamins and minerals for all sports food products and the contribution to energy of certain nutrients for a set of specific sports food categories (10). These include high carbohydrate supplements (contains between 15 and 90% average energy content from carbohydrate), protein energy supplements (contains between 15 and 30% average energy content from protein and not more than 25% from fat), and energy supplements (contains not more than 20% average energy content from protein) (10). Furthermore, in the Australian context, sports foods are required to display a warning statement to indicate they are not suitable for children under 15 years or pregnant women and that they must be consumed under medical or dietetic supervision. They must also provide consumption recommendations and restriction guides for safe intake (10). Sports foods are intended for athletes and may be recommended in some instances. They are not meant for consumption by the general non-athlete population, however, their sale in mainstream retail outlets make them more available to this population and may normalise their consumption for those who do not need the additional nutrients.

Previous Australian population surveys suggest that some non-athlete consumers could be consuming sports foods unnecessarily, incorrectly or in a harmful manner (12, 13). From an Australian public health perspective this is concerning as these foods include nutrients not deficient in the population (14), a large array of added sweeteners, and could lead to over consumption of energy, added sugars or sodium, thereby exacerbating existing diet related chronic disease risk (14). Despite their speciality nature, these products are available for sale in mainstream retail outlets such as supermarkets and pharmacies, making them easily available to the general public (15). The retail sales value of these products in Australia has increased by 195% since 2011 and at the same time availability across mainstream retail has also increased (15). It is likely, therefore, that a proportion of these sales are to non-athlete consumers who shop in mainstream retail environments. Similar trends in sales and availability have been observed in other countries, such as the United Kingdom, Eastern and Western Europe, United States, Canada, and throughout the world (16–21).

An Australian study conducted in 2013, which examined consumer use of sports foods, found that product labels such as claims and marketing statements on-pack were the most frequently used consumer sources of information to determine the risks and benefits of consumption (12). Sports food packaging in Australia can display a range of claims and marketing statements, many of which are not covered by the FSC and hence are not as strongly regulated as those that are covered by the code. With many different claims able to be displayed, the general population may find it difficult to ascertain the appropriateness of the product for their needs, or whether the product is safe to consume. To date, there have been no product audits conducted to our knowledge which examine the number and types of sports foods being sold in mainstream retail environments. Limited research which examines packaging attributes and how these foods are being marketed *via* on pack attributes (22), even though they are important in influencing consumption (23–26) and the nutrients/ingredient composition (22, 27), however, this research examines either only protein dominant products, or is based on a chemical analysis of the composition of these foods. Other existing research focuses on patterns of consumption by non-athletes, however, were conducted over 9 years ago and are therefore now of limited relevance due to the rapid expansion in the sports food market (12, 13, 27).

Detailed understanding of the current availability, product ranges, stated composition, ingredients such as sweeteners and the display of packaging attributes on these products, is important for determining if current food regulatory measures are fit for purpose. The objectives of this study were to conduct a visual analysis of the sports food products available in mainstream retail environments and to investigate the total number and types of products available in stores, the stated nutrient composition, the addition of sweeteners and the nature, number and type of written claims and marketing statements displayed on the packaging. We hypothesise that there will be multiple products with differing nutritional attributes, sweeteners will be used widely and there will be numerous claims on labels.

## Materials and methods

### Study design

This study was a retail product audit of sports foods located in store based retailers such as supermarkets, pharmacies, health food stores and gyms, these were chosen over online retailers as the majority of sports food sales (61.5%) are made in these locations in Australia (15). This study involved no human participants, and therefore ethical approval was not required.

### Data collection

Data were collected from 18 stores throughout Melbourne, Victoria, Australia, between March 2021 and May 2021. The two largest or flagship store-based retailers (communication direct from companies—3rd–5th February 2021), were chosen to increase the likelihood that the most comprehensive product variety would be available. These were: Coles, Woolworths, SUPA IGA, ALDI, COSTCO which make up 85% total grocery market share in Australia (28); The My Chemist Retail Group (Chemist Warehouse), which has the largest (20.6%) pharmacy market share (29); The health food retailer GoVita which consist of 22.9% of store based retail locations for sports foods in Australia (15); and Goodlife health club, and Fitness First gym facilities, both owned by the Fitness and Lifestyle Group, constituting 28.2% of the gym and fitness centre market share in Australia (30).

In-store data were collected using a smart phone device to capture all sides of the product packaging which were subsequently compiled into a database by researcher CC. Images of items unavailable at the time of the audit or those with illegible labels were collected from company websites or the Mintel Global New Products Database. All data were manually entered into a Microsoft Excel spreadsheet for further analysis. As per previous research (31), a random sample of 10% of products was extracted and examined by a second researcher (JW) to ensure accuracy of data entry and where discrepancies arose, agreement was reached *via* discussion and products were excluded when they did not meet the criteria.

Extracted data included: product brand, product name, flavour/variety, sports food product category (namely protein, carbohydrate dominant, other sports food), store name, aisle location (namely sports and diet, sports nutrition, health food, healthy living aisles), presence/absence of prescribed name/warning statement, nutrition information for energy, protein, fat, saturated fat, carbohydrate, sugars, dietary fibre (when displayed as it is not mandatory to display this unless a claim about fibre is made) and sodium per 100 g, whether the ingredients list was displayed, presence of sweeteners by name or code from ingredients list, serving size, pack size, serves per pack, price in AUD (unit price), and numbers of the following types of claims: nutrition content claims (e.g., high protein, 30 g protein); general level health claims (nutrient and physiological function relationship, e.g., protein for increased muscle mass); high level health claims (nutrient and disease relationship); sports effect claims (effect of nutrient on sports participation or sports outcome, e.g., bulk, shred, recover); product quality (premium, high quality); no/free from (no additives, preservatives, colours, flavours, and/or free from dairy, hormones and/or additives); taste (tastes great, delicious); natural (all natural, natural energy); sporting/organisational (trusted by sport, HACCP tested, guaranteed); vegan/vegetarian/plant-based (vegan, vegetarian, made from plants, plant-based); dieting/weight loss (slim, lose weight, diet); organic (certified organic, organic ingredients); diet style keto/paleo (100% keto, paleo friendly); health star rating and; daily intake guide. The FSC Standard 1.2.7 was used to classify regulated nutrition content and health claims (32) and additional claim classifications were determined after extensive inspection of the product packaging and a nomenclature was developed based on the type of message that each claim was conveying. Where products were available in multiple flavours, each flavour was identified and counted as a separate product. Products were only counted once even if the same product was available in multiple stores. However, each store the products were available in was recorded separately, to capture the true availability.

### Product categorisation

All products that resembled sports foods (sports food like products) located in the designated aisles of the audited stores were initially collected. Products included in the audit were products displaying either the prescribed name, “Formulated Supplementary Sports Food,” or the warning and advisory statements as required by FSC Standard 2.9.4. There were 39 products removed due to displaying the prescribed names “Supplemented food”; 19 “Formulated Supplementary food”; 38 “Dietary supplement” and 2 displaying “Formulated Caffeinated Beverage.” The remaining excluded products either did not display any prescribed name or warning/advisory statements or displayed statements that were not part of any standard such as “flavoured protein bar.” After completion of further data cleaning [determining % energy from nutrients to their energy factors, using the Nutrient Panel Calculator energy equation (protein 17 kJ/g, carbohydrate 17 kJ/g, fat 37 kJ/g, dietary fibre 8 kJ/g) (33) and comparing to stated energy in kJ per 100 g on the Nutrition Information Panel (NIP)], it became clear that some of these products did not appear to have an accurate NIP and so a further criterion of nutrients within 5% of calculated energy content based on stated nutrient content was applied. These differences may be due to the use of different energy factors for all nutrients including available carbohydrate and the inclusion or exclusion of other energy yielding substances (33). The products containing sugar alcohols only had these listed in the ingredients list and as such, their contribution to energy content could not be calculated. All products that were correctly identified as sports foods *via* the prescribed name or warning were included in the frequency and labelling results, but only those meeting the % energy criteria were included in the nutritional analysis and comparison.

Products were further identified as meeting the nutrient criteria for three additional specific categories (high carbohydrate, protein energy and energy supplement), identified in Standard 2.9.4 of the FSC. However, as very few (*n* = 9) products met these criteria, it was determined that the best way of categorising the products was as follows: protein dominant powder, protein dominant RTD shake, protein dominant bar/snack, carbohydrate dominant powder/gel, and other sports food product.

### Data analysis

Data were analysed using the Statistical Package for the Social Sciences (SPSS for Macintosh) version 28.0.1.0 (SPSS Inc., Chicago, IL, USA). Tests for normality were conducted on all data, which were not normally distributed. Descriptive statistics were used to examine the median, interquartile range, and minimum/maximum ranges of nutrients per serving suggestion within each sports food category; the number of sweeteners added to each product and the most frequently used sweeteners. A Kruskal Wallis test for medians was used to determine statistical differences in nutrient composition between sports food categories. The claim frequency data were not normally distributed, however, the mean, standard deviation, and minimum/maximum claim frequency for all products and for each of the sports food categories was used. This was due to the mean and standard deviation providing a more meaningful interpretation of the data. Additionally, significance testing could not be conducted as differences in package size between the categories was a contributing factor in how many claims could be displayed.

## Results

### General characteristics

**FIGURE 1:**
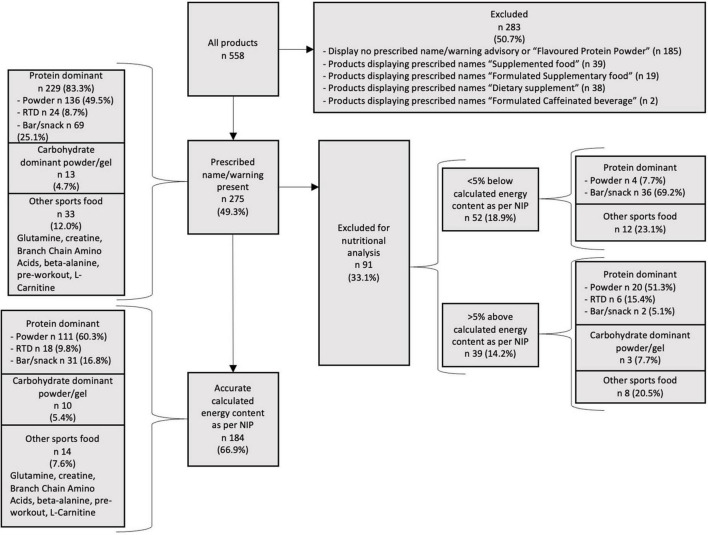
Product categorisation and products per sports food category.

There were 558 products captured during the audit, with 283 being excluded for not displaying the prescribed name “Formulated Supplementary Sports Food (FSSF)” and/or warning and advisory statements. There were 275 products in the final data set for the packaging attribute and sweetener analyses, with 83.3% being protein dominant (49.5% powders, 8.7% RTD beverages, and 25.1% bars/snacks). Only 4.7% of products were carbohydrate dominant (powders, gels) and 12.0% were other sports food products. Only 184 products (66.9%) appeared to have a sufficiently accurate NIP values as to fall within 5% of calculated energy content from protein/carbohydrate/fat/fibre, with 60.3% protein dominant powders, 9.8% protein dominant RTD beverages, 16.8% protein dominant bars/snacks, 5.4% carbohydrate dominant (powders, gels), and 7.6% other sports food products (Figure 1). Of the products which appeared to have an accurate NIP detail that met the specific compositional categories outlined by the Standard 2.9.4, only 6 (4.1%) met the protein energy criteria, all carbohydrate products met the high carbohydrate criteria and 3 (20%) met the energy criteria.

Chemist Warehouse had the largest selection of sports food products available, with 47.6% available in these locations, followed by GoVita and Coles which had 25.8 and 24.7% available, respectively. SUPA IGA and Woolworths had 19.6 and 18.2% of products available in these locations. The remaining stores ALDI, COSTCO, and Goodlife/Fitness First had the least products available in these locations.

### Nutritional characteristics

The following data represents only those products whose NIP calculations appeared to be accurate (*n* = 184). Table 1 outlines the nutrient content of the major sports foods categories. For all categories there were large variations in nutrient content, particularly in the energy, fat, saturated fat, dietary fibre, and sodium content. In relation to comparisons between protein categories, protein dominant bars/snacks had significantly higher (*p* < 0.05) median energy (2261 kJ), significantly higher (*p* < 0.01) total sugars (3.6 g) and significantly higher (all *p* < 0.001) median fat (19.8 g), saturated fat (7.2 g), carbohydrate (14.4 g), dietary fibre (12.0 g), and sodium (504.0 mg) per serving suggestion, compared to protein powders and RTD protein shakes. Carbohydrate dominant and other sports food products were similarly highly variable in the nutrients they contained. Other Sports foods were less variable, generally containing only one or two ingredients and very few nutrients apart from small amounts of energy (Table 1).

**TABLE 1:** The median nutrient content per recommended servings per day of 184 sports foods with sufficiently accurate energy content as stated on the NIP, between different categories of formulated supplementary sports foods, and the significant difference between nutrients per serving suggestion of protein dominant foods.

Nutrient per recommended servings per day	Protein powder (*n* = 111)			Protein RTD (*n* = 18)			Protein bar/snack (*n* = 31)			Carbohydrate powder/gel (*n* = 10)			Other sports food (*n* = 14)		
**Median IQR Min-Max**															
Energy (kJ)	1008	971	265–4779	741	811	554–2070	2261*	1424	680–3293	589	3489	495–13440	33	72	12–318
Protein (g)	44.3	41.1	15.0–102.1	29.3	44.1	17.3–70.3	44.3	46.4	8.8–63.0	–	–	–	1.5	1.4	0.0–14.6
Fat (g)	2.6	4.2	0.0–30.2	3.1	2.1	2.1–5.0	19.8***	11.3	6.1–60.5	–	0.1	0.0–0.1	–	–	0.0–0.7
Saturated fat (g)	1.5	2.1	0.0–23.5	1.9	1.5	0.0–2.3	7.2***	8.3	1.0–38.6	–	–	–	–	–	0.0–0.7
Carbohydrate (g)	5.5	6.7	0.0–131.6	5.7	12.0	2.3–50.3	14.4***	10.3	5.0–78.5	34.7	201.1	29.8–768.0	–	2.5	0.0–9.8
Total sugars (g)	2.2	4.9	0.0–56.4	2.1	7.0	0.1–43.5	3.6**	14.1	1.5–46.3	13.7	113.2	2.9–384.0	–	0.4	0.0–2.9
Dietary fibre (g)	0.3	2.0	0.0–7.3	0.3	2.4	0.0–6.8	12.0***	37.3	0.0–50.4	–	–	–	–	–	0.0–4.1
Sodium (mg)	162.4	250.4	0.2–1262.3	421.2	149.7	230.8–750.0	504.0***	511.8	92.0–811.8	36.0	601.7	17.5–1603.2	–	3.8	0.0–789.1

*p < 0.05, **p < 0.01, ***p < 0.001 Kruskal–Wallis test for medians across protein dominant products.

### Sweeteners

Across all products (*n* = 275), there were 19 different sweetener types identified in the ingredients list and these ranged from basic mono- and disaccharides (e.g., glucose, fructose, sucrose) to more novel sweeteners such as steviol glycosides (stevia), erythritol, and monk fruit, and non-nutritive sweeteners such as acesulphame potassium. There were 38.2% of products that contained only 1 sweetener, 34.9% contained 2 sweeteners, 12.0% contained 3 and 8.7% contained 4 or more sweeteners. The most prolific sweetener was steviol glycosides found in 44.4% of the sports foods, followed by sucralose (39.3%) and maltodextrin found in 22.9% of the sports foods identified. Only 17 (6.2%) products did not contain any sweeteners, the majority of these (11 products) were other sports foods. Products with 2 or more sweeteners were likely to be protein powders, RTDs, and bars.

For the products which had accurate calculated energy content, only 1 product contained both saccharin and cyclamate. The most prolific sweeteners were sucralose (46.1%), stevia (31.1%), and maltodextrin (28.7%). In the sports foods with calculated energy content below what was stated on the NIP, stevia was the most prolific (65.8%), followed by maltitol (35.4%), and erythritol (31.6%). The sports foods with calculated energy content above also contained stevia as the most prolific sweetener (62.1%), followed by maltodextrin (34.5%) and sucralose (24.1%).

There were minimal differences in the number of sweeteners used between the products with accurate and those that appeared to have inaccurately calculated energy content. Sports foods with below the calculated energy content were more likely to contain 2 sweeteners per product (35.4%) and had the most products which contained 5 sweeteners (21.5%), compared to the accurate products which were more likely to contain one sweetener (46.7%) and only one sports food contained 5 sweetener types in the one product.

### Percentage of items displaying a claim, claims per item, and claims per product category

All the audited sports foods displayed multiple claims or marketing statements on the packaging. The most prevalent claims were nutrition content (on 98.5% of products), general level health (65.1% of products), sports effect (62.2% of products), and product quality claims (52.7% of products). The highest number of claims on any pack was 67 which was on an “other sports food” product containing electrolytes, magnesium, and branched chain amino acids. Protein dominant powders displayed the highest mean number of claims per pack (*M* = 25.3 ± 13.1, range 1–57), closely followed by other sports foods (*M* = 24.7 ± 15.2, range 2–67). There were no high-level health claims displayed on any of the products. For protein dominant powders (*M* = 9.4 ± 5.0, range 1–24), RTD shakes (*M* = 10.6 ± 3.4, range 4–17), and other sports foods (*M* = 7.0 ± 4.2, range 0–20) nutrition content claims were the most prolific. Carbohydrate dominant gels had a higher (*M* = 2.9 ± 3.0, range 0–8) mean number of general level health claims. Carbohydrate dominant products displayed the smallest mix of claims with only 6 different types of claims compared to most other categories with 9–13 different types of claims (Table 2).

**TABLE 2:** Total 275 sports food products displaying packaging claim categories and descriptive statistics of packaging claim types per sports food category.

Claim category	All products n (%)		Protein dominant powder (*n* = 136)			Protein dominant RTD (*n* = 24)			Protein dominant bar/snack (*n* = 69)			Carbohydrate dominant powder/gel (*n* = 13)			Other sports food (*n* = 33)		
	n	%	Mean SD Min-Max														
All claim categories	275	100	25.3	13.1	1–57	19.5	5.8	9–26	11.3	5.5	3–23	8.0	3.7	5–14	24.7	15.2	2–67
Nutrition content	271	98.5	9.4	5.0	1–24	10.6	3.4	4–17	6.2	2.7	3–14	1.9	0.7	0–3	7.0	4.2	0–20
Health—General	179	65.1	4.1	4.3	0–18	0.8	1.2	0–4	1.7	2.8	0–8	2.9	3.0	0–8	6.3	5.5	0–18
Health—High	–	–	–	–		–	–	–	–	–	–	–	–	–	–	–	–
Sports effect	171	62.2	2.6	3.1	0–13	1.9	2.0	0–7	0.6	1.2	0–6	2.2	1.5	1–5	2.6	2.5	0–9
Dieting/Weight loss	35	12.7	0.5	1.2	0–9	–	–	–	0.1	0.2	0–1	–	–	–	0.1	0.2	0–1
Product quality	145	52.7	2.2	2.6	0–11	1.6	1.6	0–5	0.3	0.5	0–1	–	–	–	0.9	1.3	0–6
Taste	109	39.6	0.8	0.9	0-3	0.8	0.9	0–2	0.3	0.7	0–3	0.2	0.4	0–1	0.2	0.4	0–1
No/Free from	120	43.6	1.5	2.3	0-11	1.0	1.6	0–4	1.0	1.5	0–8	–	–	–	2.4	3.4	0–13
Natural	96	34.9	0.9	1.3	0-9	0.3	0.4	0–1	0.2	0.5	0–2	0.3	0.5	0–1	1.6	2.7	0–10
Organic	25	9.1	0.5	2.1	0–13	–	–	–	0.1	0.5	0–2	0.5	1.0	0–3	0.1	0.2	0–1
Sporting/Organisation	90	32.7	1.3	2.0	0-8	0.3	0.5	0–1	0.2	0.6	0–2	–	–	–	1.0	1.1	0–3
Vegan/Plant based	87	31.6	1.3	2.2	0-7	2.0	3.7	0–9	0.5	1.1	0–3	–	–	–	1.9	2.5	0–8
Diet style—keto/paleo	25	9.1	0.4	1.6	0-9	–	–	–	0.0	0.2	0–1	–	–	–	0.8	2.5	0–11

## Discussion

To our knowledge, this is the first product audit conducted on all sports food types in these retail locations. This study aimed to determine the availability of sports foods in Australian mainstream retail, including the types of products available, the nutrient and sweetener content and the number, type and frequency of claims displayed on the packaging. The key findings suggest that numerous sports food like products are available in mainstream retailers, however, just under half (49.3%) of these were actual FSSF. Other key findings were the appearance of inaccuracy in the calculated energy content of fat, carbohydrate, protein, and dietary fibre, stated on the NIP, the variation in nutrient content within sports food categories, the prolific use of multiple sweeteners, and the vast number of claims displayed on the packaging.

This study demonstrated that there are a large number of sports food like products (*n* = 558) located in the designated aisles, currently sold in Australian mainstream retail environments. The sports foods (*n* = 283) that were technically not FSSF (i.e., they did not meet the criteria set by FSANZ) were visually comparable, contained similar ingredients, displayed many of the same claims and could therefore be confused by consumers to be genuine sports foods. Given their availability in local retail outlets, it is likely that these products are not only being purchased by the target market (athletes) but are being purchased by the general (non-athlete) population. The sheer number and variety of products in mainstream retail environments (whether they are true FSSF or not) suggests that non-athlete consumers may be being misled and deceived, purchasing products that may or may not meet their needs. This suggests that a stronger approach to regulation may be needed, to either clearly differentiate products or potentially restrict where these foods can be purchased from. It also suggests that with such a large number of sports food like foods on the market, the time is ripe to apply further regulatory oversight.

Just under half (49.3%, *n* = 275) of the sports foods could be classified as FSSF by virtue of being labelled with the prescribed name and warning advisory statements. The presence of warnings or advisory statements, which could guide selection and use, were generally located on the rear of the packaging in small font and previous research has found that consumers are generally unaware of their presence, or meaning (13). This important information is usually overlooked during the selection process (13) and consumers are therefore less likely to appreciate the associated consumption risks and to know whether these products are beneficial for them. This could lead to consumption of products containing nutrients or substances that consumers do not need and/or in harmful quantities (34), potentially exacerbating diet related disease (35).

A surprising finding of this study, with potentially serious regulatory consequences, was the discrepancy between the energy (kJ) from the nutrients listed on the NIP and the stated energy content. Many of the products appeared to display inaccurate calculated average energy content (33.1%) which was either above (14.2%) or below (18.9%) the stated figure, although not all products (*n* = 111) displayed dietary fibre on the NIP, so these figures may change if energy from fibre could have been calculated. The NIP is an important on-pack attribute which provides the consumer with details about the nutrients present in food and what is contained in a serving of the product. Standard 1.2.8 of the FSC states that all packaged food (besides a specified list of items), must display a NIP that provides consistent and accurate information on serving size, servings per package, quantity of macro and micronutrients per 100 g and per serving size (36). There is also Australian Consumer Law that states “A person must not, in trade or commerce, engage in conduct that is misleading or deceptive or is likely to mislead or deceive” [(37), p. 104] with potential criminal charges for non-compliance.

Protein bars were the category that appeared to display inaccurate NIPs most frequently (69.2%), with lower calculated energy content ranging from 73.5 to 93.4% of stated energy content. Protein products such as bars have been identified throughout the world as the most frequently sold and consumed sports food products (12, 21), with protein bars the most frequently selected (38). It is unclear why there were so many products with false and potentially inaccurate information within the NIP, in particular those that underestimated the energy content (39). Some products lacked dietary fibre information on the NIP and the energy from sweeteners besides sugar could not be calculated, therefore it was difficult to replicate the calculation used by the manufacturer, but this is unlikely to explain all cases of inaccurate NIPs. The detection of both potentially inaccurately calculated average energy content and substantially lower energy contents of protein bars, is concerning from a consumer and public health standpoint, as consumers could be ingesting different nutrient amounts compared to what they intended. Sports foods must display an NIP which is a true and accurate reflection of the actual nutritional quality of the product (36) and the extent of this level of inaccuracy is a clear indication that some manufacturers are not practicing sufficient quality assurance processes and that enforcement agencies need to step in now and act to safeguard public health and safety. The findings also create doubt regarding the accuracy of all nutrition levels provided, the ingredients included and of the veracity of claims displayed on-pack. Analytical studies of claims relating to single ingredients in selected sports foods, concluded similarly that most of the claims made should be either modified or eliminated and could be misleading consumers (40).

This study identified a high level of variation in the dominant nutrients between each sports food category, such as protein, fat, saturated fat, carbohydrate, and dietary fibre, as has been found by other analytical studies (22, 40). This variation may be due to manufacturer determined serving size and recommended serves per day, as well as to the form of the product (e.g., powder, liquid, or bar). To account for some of these factors we only reported on the recommended servings per day for each product within each category but still identified a high level of variance despite products claiming to be sources of certain nutrients and the concomitant health or sports effects. This high level of variation in nutrients requires the consumer to be vigilant in reading all the nutrition information and serving suggestions, which consumers do not tend to do when selecting foods (41, 42). With so many different sports food products to compare, it is understandable consumers may become confused and unable to make informed choices. Consumers may reasonably expect that a sports food claiming to provide protein for instance would not provide an increased amount of fat to other protein containing products. The current Standard 2.9.4 has nutrient specifications for certain subclasses of FSSF, however, we have found here, that the majority of these foods do not fit within these sub-classes and hence there are no expected nutritional specifications for most of the sports foods on the market (10). Future consideration of these findings would assist in creating a clearer classification within the Standard, providing clear required specifications of nutrient ranges for sports food categories, or a minimum nutrient content for classification.

Nineteen different sweeteners were found in the sports foods examined in the audit ranging from mono- and di-saccharides, non-nutritive, and novel sweeteners. Interestingly, the most prolific of these were sucralose (a synthetic non-nutritive sweetener) and stevia a natural non-nutritive sweetener, derived from the leaves of the Stevia Rebaudiana, a shrub native to South America (43, 44). Both of these have a kilojoule content which is far lower than sucrose (39). Sweeteners are added to foods for a variety of reasons, to provide a sweet taste, ensure the product is palatable, and replace sensory qualities, without increasing the energy or carbohydrate content associated with regular sweeteners such as sucrose (45). Different sweeteners will have different levels of sweetness and there may be more than 1–2 different sweeteners included in the one product for palatability. Certain sweeteners are also added to provide carbohydrate content to those sports foods described as carbohydrate dominant to fuel performance. There is some controversy around non-nutritive sweeteners and health outcomes, particularly with the more novel sweeteners such as sucralose and stevia. There are observational research findings showing associations between consumption and changes to the gut microbiome (43), weight gain (46), and an increased risk of type-two diabetes (47). In this study, there was found to be minimal difference between the inclusion of sweeteners and the accuracy of calculated energy content as per the NIP and it was observed that sweeteners were contained in most sports foods and in some cases multiple in one product. Yet, packaged foods are not required to display the nutrient content in grams on the NIP, they are only required to state if they are contained in the product *via* the ingredients list. The type and amount of sweeteners used, is most likely dependant on the type of product such as a powder vs. a bar. Whether these are exceeding the upper limits is unknown, as are the health effects of these sweeteners when consumed in combination.

The audited sports foods typically displayed a range of on pack claims and marketing statements. The most frequently displayed on-pack attributes were nutrition content claims (98.5%) and general level health claims (65.1%), which is expected given the nature of these foods is to enhance some aspect of physical performance or health. However, 62.2% of products also displayed “sports effects claims” (i.e., effect of nutrient on sports participation or sports outcome, e.g., bulk, shred, recover) which do not have the same regulatory oversight as nutrition and health claims. Nutrition content claims and general level health claims, are regulated by the FSC and can only be made where certain nutrient criteria are met (32). Given the level of potential inaccuracies found in relation the energy and nutrients on the NIP of many of these foods, there are implications for the veracity of these claims. A very recent sports food audit conducted in Spain, found that most of the products complied with the relevant labelling standards (22). However, the sample was smaller than in the current audit, included only protein isolate products and examined protein quality, which was not a focus of the current study. Additionally, the European Union legislative framework has no specific regulations with reference to claims for sports foods and these are categorised as foodstuffs, therefore it is difficult to establish whether these foods do in fact meet the labelling requirements (22). Claims are important to consumers and influence choice, preference, and consumption (25, 48–52), and are sources of information that are easily processed by consumers *via* heuristics (53). This process involves making decisions using fast and automatic processes which are emotional or intuitive (54). It is likely therefore that consumers may be making product choices based on false and misleading claims, or on claims and marketing statements that are minimally regulated and are hence being deceived in the process.

Another interesting finding was the vast cacophony of claims displayed on many of these foods, with a mean of around 11–25 claims per pack on most types of foods (except for carbohydrate foods which had a mean of eight claims). The sheer quantity of claims could make it difficult for consumers to process all of the information and then to make an informed choice. Studies have indicated that multiple, competing pieces of information on food packs can increase consumer confusion (23–25, 49). With the expansion of the market and so many claims per pack, which are not regulated in the same way as nutrition and health claims, it is timely to consider further regulatory changes that would reduce the messaging load on consumers and enhance their decision-making processes.

### Strengths and limitations

This study had several strengths. Due to the comprehensive number of stores that were audited and the collection of information from the two largest stores of each retailer, it is likely that the majority of products available in mainstream retailers within Australia were captured. To our knowledge, this is the first study of this kind undertaken in Australia and can add considerably to regulatory decisions currently under investigation. Furthermore, this study provides a novel, comprehensive classification system for the on-pack attributes displayed on sports foods, which has not previously existed and can be used as a baseline for future research in this area.

The limitations of this study include the use of a cross-sectional methodology, which depicts the products available at only one point in time. It also relies on the nutrition information printed on the packaging, which did not include all nutrients in some cases, which makes it difficult to fully replicate the manufacturers calculation for all products. Therefore, a large number of products appeared to be inaccurate in this study. Additionally, this study only examined products sold at mainstream physical stores in Australia and not products sold through digital retailers located online (comprising 38.5% of the retail market in Australia). As no online stores were included in this audit, it is possible that a sector of the sports food market was missed. This could be mitigated, in future by conducting a comprehensive audit which also includes digital retailers.

## Conclusion

It is vitally important that consumers are provided accurate and detailed on-pack information regarding nutrition content, ingredients, additives, claims, and potential warnings about the foods they select. The present study showed that there were a large number of sports food like products being sold in mainstream retail markets. Just over half (50.7%) did not conform with the current display of required statements for formulated supplementary sports foods, contained multiple non-nutritive sweeteners, displayed an overwhelming number of claims and approximately 33% of sports food products appeared to have inaccurate nutrition information. The current Standard was published (gazetted) in 2001 and is clearly outdated. Considering the expansion in the sales, the large number of products and their availability in mainstream retail, this is of concern and could impact not only the intended market (athletes) but also the general non-athlete consumer. The results indicate potentially underperforming manufacturing processes that preference marketing over product quality and call for a strengthening of regulatory approaches to protect consumer health and safety and prevent consumer deception.

## Data availability statement

The raw data supporting the conclusions of this article will be made available by the authors, without undue reservation.

## Author contributions

CC and JW conceptualised the study and analysed the data. CC conducted the data collection. JW, CR, and AB provided input into the study design and methods and contributed to writing and editing. JW provided input into the data analysis and interpretation and conducted the data checks. All authors read and approved the final manuscript.

## References

[1] Department of Health & Human Services. *Sporting Performance and Food State of Victoria.* Melbourne, VIC: State Government of Victoria (2020).

[2] Australian Institute of Sport. *Nutrition.* (2022). Available online at: https://www.ais.gov.au/nutrition (accessed June 22, 2022).

[3] Australian Institute of Sport. *Supplements: The Benefits and Risks of Using Supplements and Sports Foods.* (2022). Available online at: https://www.ais.gov.au/nutrition/supplements (accessed June 22, 2022).

[4] Australian institute of sport. *The Ais Sports Supplement Framework.* (2021). Available online at: https://www.ais.gov.au/nutrition/supplements (accessed August 13, 2022).

[5] Food Standards Agency. *Food Supplements.* (2021). Available online at: https://www.food.gov.uk/business-guidance/food-supplements#what-a-food-supplement-is (accessed August 4, 2022).

[6] European Union. *Directive 2009:39:Ec of the European Parliment Ond of the Council.* (2009). Available online at: https://eur-lex.europa.eu/legal-content/EN/TXT/?uri=CELEX%3A32009L0039&qid=1659576097417 (accessed August 4, 2022).

[7] National Institutes of Health Office of Dietary Supplements. *Dietary Supplements for Exercise and Athletic Performance.* Bethesda, MD: Office of Dietary Supplements (2021).

[8] Government of Canada. *About Supplemented Foods and Thier Labels.* (2022). Available online at: https://www.canada.ca/en/health-canada/services/food-nutrition/supplemented-foods/about.html (accessed August 4, 2022)

[9] European Union. *Corrigendum to Regulation (Ec) No 1924:2006 of the European Parliament and of the Council of 20 December 2006 on Nutrition and Health Claims Made on Foods.* (2006). Available online at: https://eur-lex.europa.eu/eli/reg/2006/1924/oj (accessed August 4, 2022).

[10] FSANZ. *Food Standards Code, Formulated Supplementary Sports Food.* (2020). Available online at: https://www.legislation.gov.au/Series/F2015L00421 (accessed July 1, 2020).

[11] Martinez-Sanz J Sospedra I Baladia E Arranz L Ortiz-Moncada R Gil-Izquierdo A. Current status of legislation on dietary products for sportspeople in a European framework. *Nutrients.* (2017) 9:1225. 10.3390/nu9111225 29117104 PMC5707697

[12] Food Standards Australia New Zealand. *Sports Foods Consumption in Australia and New Zealand.* Food Standards Australia New Zealand (2013).

[13] Food Standards Australia New Zealand. *Consumer Research Investigating the Use of Formulated Supplementary Sports Foods* (2010).

[14] ABS. *National Health Survey: First Results, 2017-18.* (2017-2018). Available online at: https://www.abs.gov.au/ausstats/abs@.nsf/Lookup/by%20Subject/4364.0.55.001~2017-18~Main%20Features~Overweight%20and%20obesity~90 (accessed March 20, 2019).

[15] Euromonitor International. *Sports Nutrition in Australia.* London: Euromonitor International (2021).

[16] Euromonitor International. *Sports Nutrition in the United Kingdom.* London: Euromonitor International (2022).

[17] Euromonitor International. *Sports Nutrition in Eastern Europe Datagraphic.* London: Euromonitor International (2022).

[18] Euromonitor International. *Sports Nutrition in Western Europe.* London: Euromonitor International (2022).

[19] Euromonitor International. *Sports Nutrition in the United States of America.* London: Euromonitor International (2022).

[20] Euromonitor International. *Sports Nutrition in Canada.* London: Euromonitor International (2022).

[21] Euromonitor International. *Sports Nutrition in World.* London: Euromonitor International (2021).

[22] Rodriguez-Lopez P Rueda-Robles A Sanchez-Rodriguez L Blanca-Herrera R Quirantes-Pine R Borras-Linares I Analysis and screening of commercialized protein supplements for sports practice. *Foods.* (2022) 11:3500. 10.3390/foods11213500 36360118 PMC9658000

[23] Talati Z Pettigrew S Hughes C Dixon H Kelly B Ball K The combined effect of front-of-pack nutrition labels and health claims on consumers’ evaluation of food products. *Food Qual Prefer.* (2016) 53:57–65. 10.1016/j.foodqual.2016.05.016

[24] Gorski Findling M Werth P Musicus A Bragg M Graham D Elbel B Comparing five front-of-pack nutrition labels’ influence on consumers’ perceptions and purchase intentions. *Prev Med.* (2018) 106:114–21. 10.1016/j.ypmed.2017.10.022 29066375 PMC5764801

[25] Acton R Hammond D. Do Manufacturer ‘nutrient claims’ influence the efficacy of mandated front-of-package labels? *Public Health Nutr.* (2018) 21:3354–9. 10.1017/s1368980018002550 30345943 PMC10260817

[26] Ni Mhurchu C Eyles H Jiang Y Blakely T. Do nutrition labels influence healthier food choices? Analysis of label viewing behaviour and subsequent food purchases in a labelling intervention trial. *Appetite.* (2018) 121:360–5. 10.1016/j.appet.2017.11.105 29191745

[27] LGC. *Australian Supplement Survey.* Teddington: LGC (2016).

[28] IBIS world. *Supermarkets and Grocery Stores in Australia.* Los Angeles, CA: IBIS world (2022). p. G4111.

[29] IBIS world. *Pharmacies in Australia.* Los Angeles, CA: IBIS world (2020).

[30] IBIS world. *Gyms and Fitness Centres in Australia.* Los Angeles, CA: IBIS world (2022). p. R9111.

[31] McCann J Russell C Campbell K Woods J. Nutrition and packaging characteristics of toddler food and milks in Australia. *Public Health Nutr.* (2020) 24:1153–65. 10.1017/S1368980020004590 33183396 PMC10195549

[32] FSANZ. *Food Standards Code, Nutrition, Health and Related Claims.* (2020). Available from: https://www.legislation.gov.au/Series/F2015L00394 (accessed July 1, 2020).

[33] Food Standards Australia New Zealand. *Nutrients in the Npc.* (2020). Available online at: https://www.foodstandards.gov.au/industry/npc/Pages/Nutrients-in-the-NPC.aspx#energy (accessed September 10, 2020).

[34] Duiven E van Loon L Spruijt L Koert W de Hon O. Undeclared doping substances are highly prevalent in commercial sports nutrition supplements. *J Sports Sci Med.* (2021) 20:328–38. 10.52082/jssm.2021.328 34211326 PMC8219275

[35] Housman J Dorman S. Dietary and sports supplements the need for a systematic tracking system. *Am J Health Stud.* (2008) 23:35–40.

[36] FSANZ. *Food Standards Code, Nutrition Information Requirements.* (2020). Available online at: https://www.foodstandards.gov.au/code/Pages/default.aspx (accessed July 10, 2020).

[37] Australian Competition and Consumer Commission. *Competition and Consumer Act 2010.* Canberra, ACT: Office of Parlimentary Counsel (2010).

[38] Harwood W Drake M. Understanding implicit and explicit consumer desires for protein bars, powders, and beverages. *J Sens Stud.* (2019) 34:e12493. 10.1111/joss.12493

[39] Food Standards Australia New Zealand. *Intense Sweeteners.* (2021). Available online at: https://www.foodstandards.gov.au/consumer/additives/Pages/Sweeteners.aspx (accessed July 10, 2022).

[40] Molina Juan L Sospedra I Perales A Gonzalez-Diaz C Gil-Izquierdo A Martinez-Sanz J. Analysis of health claims regarding creatine monohydrate present in commercial communications for a sample of european sports foods supplements. *Public Health Nutr.* (2021) 24:632–40. 10.1017/S1368980020005121 33468268 PMC11574825

[41] Hodgkins C Barnett J Wasowicz-Kirylo G Stysko-Kunkowska M Gulcan Y Kustepeli Y Understanding how consumers categorise nutritional labels: a consumer derived typology for front-of-pack nutrition labelling. *Appetite.* (2012) 59:806–17. 10.1016/j.appet.2012.08.014 22918174

[42] Bialkova S Grunert K van Trijp H. Standing out in the crowd: the effect of information clutter on consumer attention for front-of-pack nutrition labels. *Food Policy.* (2013) 41:65–74. 10.1016/j.foodpol.2013.04.010

[43] Ruiz-Ojeda F Plaza-Diaz J Saez-Lara M Gil A. Effects of sweeteners on the gut microbiota: a review of experimental studies and clinical trials. *Adv Nutr.* (2019) 10 (Suppl. 1):S31–48. 10.1093/advances/nmy037 30721958 PMC6363527

[44] Chattopadhyay S Raychaudhuri U Chakraborty R. Artificial sweeteners – a review. *J Food Sci Technol.* (2014) 51:611–21. 10.1007/s13197-011-0571-1 24741154 PMC3982014

[45] Russell C Dickie S Baker P Lawrence M. Does the Australian health star rating system encourage added sugar reformulation? Trends in sweetener use in Australia. *Nutrients.* (2021) 13:898. 10.3390/nu13030898 33802024 PMC7998813

[46] Fowler S Williams K Resendez R Hunt K Hazuda H Stern M. Fueling the obesity epidemic? Artificially sweetened beverage use and long-term weight gain. *Obesity.* (2008) 16:1894–900. 10.1038/oby.2008.284 18535548

[47] O’Connor L Imamura F Lentjes M Khaw K Wareham N Forouhi N. Prospective associations and population impact of sweet beverage intake and type 2 diabetes, and effects of substitutions with alternative beverages. *Diabetologia.* (2015) 58:1474–83. 10.1007/s00125-015-3572-1 25944371 PMC4473082

[48] Aschemann-Witzel J Hamm U. Do consumers prefer foods with nutrition and health claims? Results of a purchase simulation. *J Mark Commun.* (2010) 16:47–58. 10.1080/13527260903342746

[49] Bialkova S Sasse L Fenko A. The role of nutrition labels and advertising claims in altering consumers’ evaluation and choice. *Appetite.* (2016) 96:38–46. 10.1016/j.appet.2015.08.030 26341955

[50] Franco-Arellano B Vanderlee L Ahmed M Oh A L’Abbe M. Influence of front-of-pack labelling and regulated nutrition claims on consumers’ perceptions of product healthfulness and purchase intentions: a randomized controlled trial. *Appetite.* (2020) 149:104629. 10.1016/j.appet.2020.104629 32061707

[51] Nobrega L Ares G Deliza R. Are nutritional warnings more efficient than claims in shaping consumers’ healthfulness perception? *Food Qual Prefer.* (2020) 79:103749. 10.1016/j.foodqual.2019.103749

[52] Talati Z Pettigrew S Neal B Dixon H Hughes C Kelly B Consumers’ responses to health claims in the context of other on-pack nutrition information: a systematic review. *Nutr Rev.* (2017) 75:260–73. 10.1093/nutrit/nuw070 28371913

[53] Kahneman D. *Thinking, Fast and Slow.* New York, NY: Farrar, Straus and Giroux (2013).

[54] Chaiken S. Heuristic versus systematic information processing and the use of source versus message cues in persuasion. *J Pers Soc Psychol.* (1980) 39:752–66.

